# Crystal structure of 3-(2,5-di­meth­oxy­phen­yl)propionic acid

**DOI:** 10.1107/S2056989015007641

**Published:** 2015-04-25

**Authors:** Bernhard Bugenhagen, Yosef Al Jasem, Mariam AlAzani, Thies Thiemann

**Affiliations:** a Institute of Inorganic Chemistry, University of Hamburg, Hamburg, Germany; b Department of Chemical Engineering; c Department of Chemistry, United Arab Emirates University, AL Ain, Abu Dhabi, United Arab Emirates

**Keywords:** crystal structure, 3-(2,5-di­meth­oxy­phen­yl)propionic acid, O—H⋯O hydrogen bonding

## Abstract

In the crystal of the title compound, C_11_H_14_O_4_, the aromatic ring is almost coplanar with the 2-position meth­oxy group with which it subtends a dihedral of 0.54 (2)°, while the 5-position meth­oxy group makes a corresponding dihedral angle of just 5.30 (2)°. The angle between the mean planes of the aromatic ring and the propionic acid group is 78.56 (2)°. The fully extended propionic side chain is in a *trans* configuration with a C—C—C—C torsion angle of −172.25 (7)°. In the crystal, hydrogen bonding is limited to dimer formation *via R*_2_^2^(8) rings. The hydrogen-bonded dimers are stacked along the *b* axis. The average planes of the two benzene rings in a dimer are parallel to each other, but at an offset of 4.31 (2) Å. Within neighbouring dimers along the [101] direction, the average mol­ecular benzene planes are almost perpendicular to each other, with a dihedral angle of 85.33 (2)°.

## Related literature   

For another preparation method of the title compound, see: Anliker *et al.* (1957 ▸). For crystal structures of phenyl­propionic acids, see: Das *et al.* (2012 ▸). For the application of the title compound as a starting material for 19-norsteroidal derivatives, see: Anliker *et al.* (1957 ▸); and as a starting material for amido­ethyl­quinones, see: Bremer *et al.* (2014 ▸).
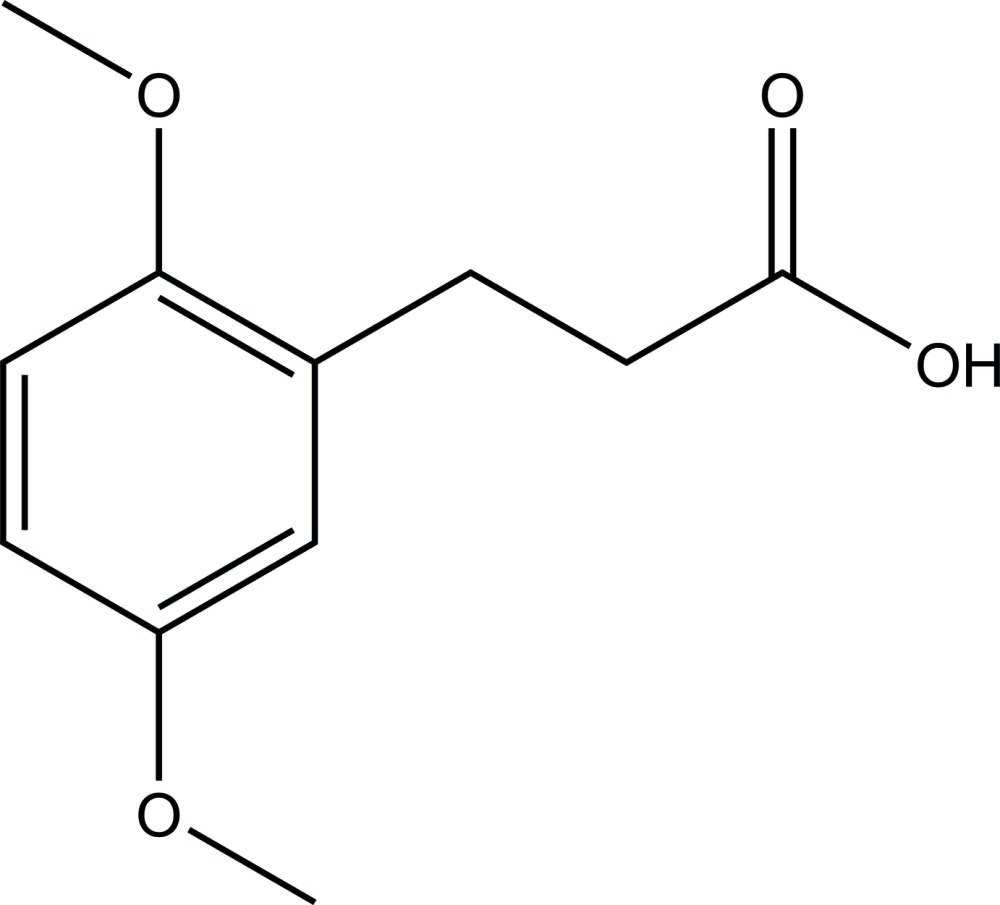


## Experimental   

### Crystal data  

C_11_H_14_O_4_
*M* *_r_* = 210.22
Monoclinic, 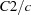

*a* = 24.3212 (10) Å
*b* = 4.6512 (2) Å
*c* = 19.7411 (8) Å
β = 109.1782 (6)°
*V* = 2109.23 (15) Å^3^
*Z* = 8
Mo *K*α radiation
μ = 0.10 mm^−1^
*T* = 100 K
0.3 × 0.1 × 0.02 mm

### Data collection  

Bruker APEXII CCD diffractometer
Absorption correction: multi-scan (*SADABS*; Bruker, 2013 ▸) *T*_min_ = 0.604, *T*_max_ = 0.746
20284 measured reflections
3224 independent reflections
2927 reflections with *I* > 2σ(*I*)
*R*_int_ = 0.028

### Refinement  

*R*[*F* ^2^ > 2σ(*F* ^2^)] = 0.036
*wR*(*F* ^2^) = 0.107
*S* = 1.05
3224 reflections
142 parameters
H atoms treated by a mixture of independent and constrained refinement
Δρ_max_ = 0.44 e Å^−3^
Δρ_min_ = −0.18 e Å^−3^

### 

Data collection: *APEX2* (Bruker, 2013 ▸); cell refinement: *SAINT* (Bruker, 2013 ▸); data reduction: *SAINT*; program(s) used to solve structure: *SIR2004* (Burla *et al.*, 2007 ▸); program(s) used to refine structure: *SHELXL2013* (Sheldrick, 2015 ▸); molecular graphics: *PLATON* (Spek, 2009 ▸) and *Mercury* (Macrae *et al.*, 2008 ▸); software used to prepare material for publication: *OLEX2* (Dolomanov *et al.*, 2009 ▸).

## Supplementary Material

Crystal structure: contains datablock(s) I. DOI: 10.1107/S2056989015007641/qm2110sup1.cif

Structure factors: contains datablock(s) I. DOI: 10.1107/S2056989015007641/qm2110Isup2.hkl

Supporting information file. DOI: 10.1107/S2056989015007641/qm2110Isup3.cml

**Fig. 1.**
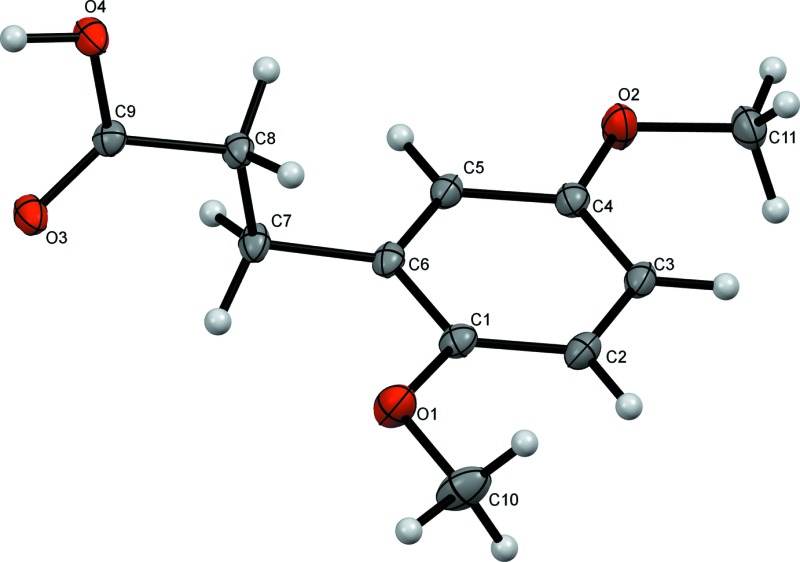
A view of title compound molecule with the atom-numbering scheme. Displacement ellipsoids are shown at the 50% probability level.

. DOI: 10.1107/S2056989015007641/qm2110fig1.tif
A view of title compound mol­ecule with the atom-numbering scheme. Displacement ellipsoids are shown at the 50% probability level.

CCDC reference: 1060285

Additional supporting information:  crystallographic information; 3D view; checkCIF report

## Figures and Tables

**Table 1:** Hydrogen-bond geometry (, )

*D*H*A*	*D*H	H*A*	*D* *A*	*D*H*A*
O4H4O3^i^	0.92(2)	1.75(2)	2.6624(11)	172.1(18)

Symmetry code: (i) 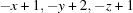.

## supplementary crystallographic information

### S1. Structural commentary

The molecule of the title compound exhibits one conformation (Figure 1), unlike other analogous compounds that exhibit two conformations (e.g. 3-phenyl­propionic acid, 3-(3-methyl­phenyl)­propionic acid and 3-(3-meth­oxy­phenyl)­propionic acid) (Das *et al.*, 2012). The aromatic ring of the title compound is almost coplanar with the C10 methoxyl with which it has a dihedral of less than 0.54 (2) ° while the C11 methoxyl has a corresponding dihedral of just 5.30 (2)°. The angle between the mean planes of the aromatic ring and the propionic acid group (C7, C8, C9, O3 and O4) is 78.56 (2) °. The fully extended propionic side chain is in a *trans* configuration with (C6—C7—C8—C9) torsion angle of -172.25 (2)°. The O4—H4···O3 hydrogen bonding (Table 1) of the COOH functional groups leads to dimer formation *via R*^2^_2_(8) rings. The hydrogen bonded dimers are stacked along the *b* axis. The average planes of the two benzene rings in a dimer are parallel to each other, but at an offset of 4.31 (2) Å. Within neighboring dimers along [101] direction, the average molecular benzene planes are almost perpendicular to each other, with an angle of 85.33 (2)°. No other appreciable close contacts were noticed except a very weak C3—H3···π inter­action between adjacent dimers along [101], with a bond length of 3.20 (2) Å.

### S2. Synthesis and crystallization

3-(2,5-Di­meth­oxy­phenyl)­propionic acid. - Ethyl 3-(2,5-di­meth­oxy­phenyl)­propionate (3.2 g, 13.4 mmol) in a mixture of aq. NaOH (10 w%, 30 mL) and methanol (8 mL) was heated at reflux for 12h. Then, half. conc. aq. HCl is added to the cooled solution. Thereafter, the mixture is extracted with chloro­form (3 X 15 mL). The organic phase is dried over anhydrous MgSO_4_ and concentrated *in vacuo*. The residue is filtered over a small column of silica gel (di­ethyl­ether–CHCl_3_, 1:1, v/v) to give the title compound (2.56 g, 89%) as colorless needles, mp. 339 – 340 K [Lit. mp. 339-340 K (Anliker *et al.*, 1957)]; *ν*_max_ (KBr/cm^-1^) 3500 – 2050 (bs, OH), 2955, 2835, 1699, 1504, 1449, 1430, 1307, 1281, 1182, 1127, 927, 916, 865, 795, 717, 499; *δ*_H_ (400 MHz, CDCl_3_) 2.65 (2H, t, ^3^*J* = 7.6 Hz), 2.91 (2H, t, ^3^*J* = 7.6 Hz), 6.71 (1H, dd, ^3^*J* = 8.4 Hz, ^4^*J* = 3.2 Hz), 6.75 (1H, d, ^4^*J* = 3.2 Hz), 6.76 (1H, d, ^3^*J* = 8.4 Hz), *δ*_C_ (67.8 MHz, CDCl_3_) 26.0 (CH_2_), 33.9 (CH_2_), 55.6 (OCH_3_), 55.7 (OCH_3_), 111.0 (CH), 111.6 (CH), 116.3 (CH), 129.6 (CH), 151.7 (C_quat_), 153.3 (C_quat_), 179.7 (C_quat_, CO).

### S3. Refinement

All hydrogen atoms were placed in calculated positions with C—H distances of 0.95- 0.99 Å and refined as riding with *U*_iso_(H) = x*U*_eq_(C), where x = 1.5 for methyl and x = 1.2 for all other H-atoms.

### Crystal data

**Table:**

C_11_H_14_O_4_	*D*_x_ = 1.324 Mg m^−^^3^
*M**_r_* = 210.22	Melting point = 339–340 K
Monoclinic, *C*2/*c*	Mo *K*α radiation, λ = 0.71073 Å
*a* = 24.3212 (10) Å	Cell parameters from 9914 reflections
*b* = 4.6512 (2) Å	θ = 2.3–31.2°
*c* = 19.7411 (8) Å	µ = 0.10 mm^−^^1^
β = 109.1782 (6)°	*T* = 100 K
*V* = 2109.23 (15) Å^3^	Bar, clear light colourless
*Z* = 8	0.3 × 0.1 × 0.02 mm
*F*(000) = 896	

### Data collection

**Table:**

Bruker APEXII CCD diffractometer	2927 reflections with *I* > 2σ(*I*)
φ and ω scans	*R*_int_ = 0.028
Absorption correction: multi-scan (*SADABS*; Bruker, 2013)	θ_max_ = 31.3°, θ_min_ = 1.8°
*T*_min_ = 0.604, *T*_max_ = 0.746	*h* = −35→34
20284 measured reflections	*k* = −6→6
3224 independent reflections	*l* = −27→28

### Refinement

**Table:**

Refinement on *F*^2^	Primary atom site location: structure-invariant direct methods
Least-squares matrix: full	Hydrogen site location: mixed
*R*[*F*^2^ > 2σ(*F*^2^)] = 0.036	H atoms treated by a mixture of independent and constrained refinement
*wR*(*F*^2^) = 0.107	*w* = 1/[σ^2^(*F*_o_^2^) + (0.0556*P*)^2^ + 1.4383*P*] where *P* = (*F*_o_^2^ + 2*F*_c_^2^)/3
*S* = 1.05	(Δ/σ)_max_ = 0.001
3224 reflections	Δρ_max_ = 0.44 e Å^−^^3^
142 parameters	Δρ_min_ = −0.18 e Å^−^^3^
0 restraints	

### Special details

**Table:**

Experimental. SADABS-2012/1 (Bruker,2012) was used for absorption correction. wR2(int) was 0.1419 before and 0.0438 after correction. The Ratio of minimum to maximum transmission is 0.8088. The λ/2 correction factor is 0.0015.
Geometry. All e.s.d.'s (except the e.s.d. in the dihedral angle between two l.s. planes) are estimated using the full covariance matrix. The cell e.s.d.'s are taken into account individually in the estimation of e.s.d.'s in distances, angles and torsion angles; correlations between e.s.d.'s in cell parameters are only used when they are defined by crystal symmetry. An approximate (isotropic) treatment of cell e.s.d.'s is used for estimating e.s.d.'s involving l.s. planes.

### Fractional atomic coordinates and isotropic or equivalent isotropic displacement parameters (Å^2^)

**Table:**

	*x*	*y*	*z*	*U*_iso_*/*U*_eq_	
C1	0.36178 (4)	0.50978 (18)	0.70073 (5)	0.01639 (17)	
C10	0.44839 (4)	0.3697 (3)	0.79439 (5)	0.0289 (2)	
C11	0.14931 (4)	0.2622 (2)	0.61260 (5)	0.02223 (19)	
C2	0.32768 (4)	0.32755 (19)	0.72601 (5)	0.01806 (17)	
C3	0.26774 (4)	0.30632 (18)	0.69042 (5)	0.01692 (17)	
C4	0.24216 (4)	0.47152 (18)	0.62979 (5)	0.01575 (16)	
C5	0.27657 (4)	0.65750 (18)	0.60495 (4)	0.01568 (16)	
C6	0.33612 (4)	0.67778 (17)	0.63904 (4)	0.01468 (16)	
C7	0.37340 (4)	0.86255 (18)	0.60859 (5)	0.01664 (16)	
C8	0.39755 (4)	0.67941 (18)	0.56036 (5)	0.01553 (16)	
C9	0.44172 (3)	0.82948 (18)	0.53467 (4)	0.01464 (16)	
H10A	0.4311	0.4146	0.8315	0.043*	
H10B	0.4901	0.4129	0.8124	0.043*	
H10C	0.4427	0.1653	0.7820	0.043*	
H11A	0.1506	0.3038	0.6618	0.033*	
H11B	0.1648	0.0690	0.6106	0.033*	
H11C	0.1090	0.2724	0.5803	0.033*	
H2	0.3452	0.2162	0.7679	0.022*	
H3	0.2447	0.1796	0.7077	0.020*	
H4	0.4791 (8)	0.795 (4)	0.4682 (10)	0.049 (5)*	
H5	0.2587	0.7724	0.5638	0.019*	
H7A	0.4059	0.9461	0.6481	0.020*	
H7B	0.3498	1.0224	0.5804	0.020*	
H8A	0.4156	0.5046	0.5871	0.019*	
H8B	0.3647	0.6170	0.5181	0.019*	
O1	0.42100 (3)	0.53955 (17)	0.73202 (4)	0.02384 (16)	
O2	0.18373 (3)	0.46840 (16)	0.59092 (4)	0.02300 (16)	
O3	0.46906 (3)	1.04245 (15)	0.56342 (4)	0.02097 (15)	
O4	0.44928 (3)	0.70388 (15)	0.47828 (4)	0.02040 (15)	

### Atomic displacement parameters (Å^2^)

**Table:**

	*U*^11^	*U*^22^	*U*^33^	*U*^12^	*U*^13^	*U*^23^
C1	0.0167 (4)	0.0191 (4)	0.0150 (4)	0.0013 (3)	0.0074 (3)	−0.0003 (3)
C10	0.0211 (4)	0.0448 (6)	0.0197 (4)	0.0072 (4)	0.0049 (3)	0.0065 (4)
C11	0.0206 (4)	0.0207 (4)	0.0263 (4)	−0.0063 (3)	0.0089 (3)	−0.0017 (3)
C2	0.0221 (4)	0.0190 (4)	0.0149 (4)	0.0013 (3)	0.0086 (3)	0.0031 (3)
C3	0.0218 (4)	0.0159 (4)	0.0162 (4)	−0.0015 (3)	0.0104 (3)	0.0008 (3)
C4	0.0170 (4)	0.0154 (3)	0.0162 (4)	−0.0009 (3)	0.0073 (3)	−0.0010 (3)
C5	0.0196 (4)	0.0144 (3)	0.0148 (3)	0.0004 (3)	0.0081 (3)	0.0015 (3)
C6	0.0190 (4)	0.0130 (3)	0.0155 (4)	0.0002 (3)	0.0103 (3)	−0.0010 (3)
C7	0.0201 (4)	0.0144 (3)	0.0200 (4)	−0.0012 (3)	0.0127 (3)	−0.0008 (3)
C8	0.0166 (4)	0.0159 (4)	0.0172 (4)	−0.0024 (3)	0.0099 (3)	−0.0016 (3)
C9	0.0137 (3)	0.0159 (4)	0.0160 (3)	0.0007 (3)	0.0072 (3)	−0.0003 (3)
O1	0.0167 (3)	0.0334 (4)	0.0207 (3)	0.0005 (3)	0.0053 (2)	0.0055 (3)
O2	0.0176 (3)	0.0258 (3)	0.0242 (3)	−0.0045 (2)	0.0049 (3)	0.0060 (3)
O3	0.0239 (3)	0.0205 (3)	0.0238 (3)	−0.0077 (2)	0.0150 (3)	−0.0069 (2)
O4	0.0208 (3)	0.0229 (3)	0.0233 (3)	−0.0074 (2)	0.0151 (3)	−0.0086 (2)

### Geometric parameters (Å, º)

**Table:**

C10—H10C	0.9800	C6—C5	1.3854 (12)
C10—H10B	0.9800	C7—H7B	0.9900
C10—H10A	0.9800	C7—H7A	0.9900
C11—H11C	0.9800	C8—C9	1.5019 (11)
C11—H11B	0.9800	C8—C7	1.5312 (11)
C11—H11A	0.9800	C8—H8B	0.9900
C2—C1	1.3882 (12)	C8—H8A	0.9900
C2—H2	0.9500	O1—C10	1.4299 (12)
C3—C4	1.3858 (12)	O1—C1	1.3751 (10)
C3—C2	1.3985 (12)	O2—C11	1.4278 (11)
C3—H3	0.9500	O2—C4	1.3756 (10)
C5—C4	1.3995 (11)	O3—C9	1.2238 (10)
C5—H5	0.9500	O4—H4	0.917 (18)
C6—C1	1.4085 (12)	O4—C9	1.3224 (10)
C6—C7	1.5101 (11)		
			
C1—C2—H2	119.7	C9—C8—H8B	108.6
C1—C2—C3	120.65 (8)	C9—C8—H8A	108.6
C1—C6—C7	120.43 (8)	C9—O4—H4	108.5 (11)
C1—O1—C10	117.01 (7)	H10A—C10—H10C	109.5
C2—C1—C6	120.15 (8)	H10A—C10—H10B	109.5
C2—C3—H3	120.2	H10B—C10—H10C	109.5
C3—C4—C5	119.66 (8)	H11A—C11—H11C	109.5
C3—C2—H2	119.7	H11A—C11—H11B	109.5
C4—C5—H5	119.3	H11B—C11—H11C	109.5
C4—C3—C2	119.53 (8)	H7A—C7—H7B	108.2
C4—C3—H3	120.2	H8A—C8—H8B	107.6
C4—O2—C11	116.09 (7)	O1—C10—H10C	109.5
C5—C6—C1	118.54 (7)	O1—C10—H10B	109.5
C5—C6—C7	120.93 (7)	O1—C10—H10A	109.5
C6—C7—H7B	109.8	O1—C1—C2	124.15 (8)
C6—C7—H7A	109.8	O1—C1—C6	115.71 (7)
C6—C7—C8	109.52 (7)	O2—C11—H11C	109.5
C6—C5—C4	121.46 (8)	O2—C11—H11B	109.5
C6—C5—H5	119.3	O2—C11—H11A	109.5
C7—C8—H8B	108.6	O2—C4—C5	115.98 (7)
C7—C8—H8A	108.6	O2—C4—C3	124.36 (8)
C8—C7—H7B	109.8	O3—C9—C8	124.03 (7)
C8—C7—H7A	109.8	O3—C9—O4	122.90 (8)
C9—C8—C7	114.48 (7)	O4—C9—C8	113.05 (7)
			
C1—C6—C7—C8	83.52 (9)	C5—C6—C1—C2	0.36 (12)
C1—C6—C5—C4	−1.12 (12)	C5—C6—C1—O1	179.97 (7)
C10—O1—C1—C2	0.15 (13)	C5—C6—C7—C8	−92.96 (9)
C10—O1—C1—C6	−179.45 (8)	C6—C5—C4—C3	0.97 (13)
C11—O2—C4—C5	175.08 (8)	C6—C5—C4—O2	−179.31 (7)
C11—O2—C4—C3	−5.21 (13)	C7—C8—C9—O3	19.85 (12)
C2—C3—C4—C5	−0.03 (13)	C7—C8—C9—O4	−161.63 (7)
C2—C3—C4—O2	−179.73 (8)	C7—C6—C1—C2	−176.20 (8)
C3—C2—C1—C6	0.56 (13)	C7—C6—C1—O1	3.41 (11)
C3—C2—C1—O1	−179.02 (8)	C7—C6—C5—C4	175.42 (7)
C4—C3—C2—C1	−0.73 (13)	C9—C8—C7—C6	−172.25 (7)

### Hydrogen-bond geometry (Å, º)

**Table:**

*D*—H···*A*	*D*—H	H···*A*	*D*···*A*	*D*—H···*A*
O4—H4···O3^i^	0.92 (2)	1.75 (2)	2.6624 (11)	172.1 (18)

Symmetry code: (i) −*x*+1, −*y*+2, −*z*+1.
