# Supplementary figures and images for: Crystal structure of 3-(2,5-di­meth­oxy­phen­yl)propionic acid
Source: Acta Crystallogr E Crystallogr Commun. 2015 Apr 25;71(Pt 5):o337–8. doi: 10.1107/S2056989015007641 (PMC4420092; doi:10.1107/S2056989015007641)

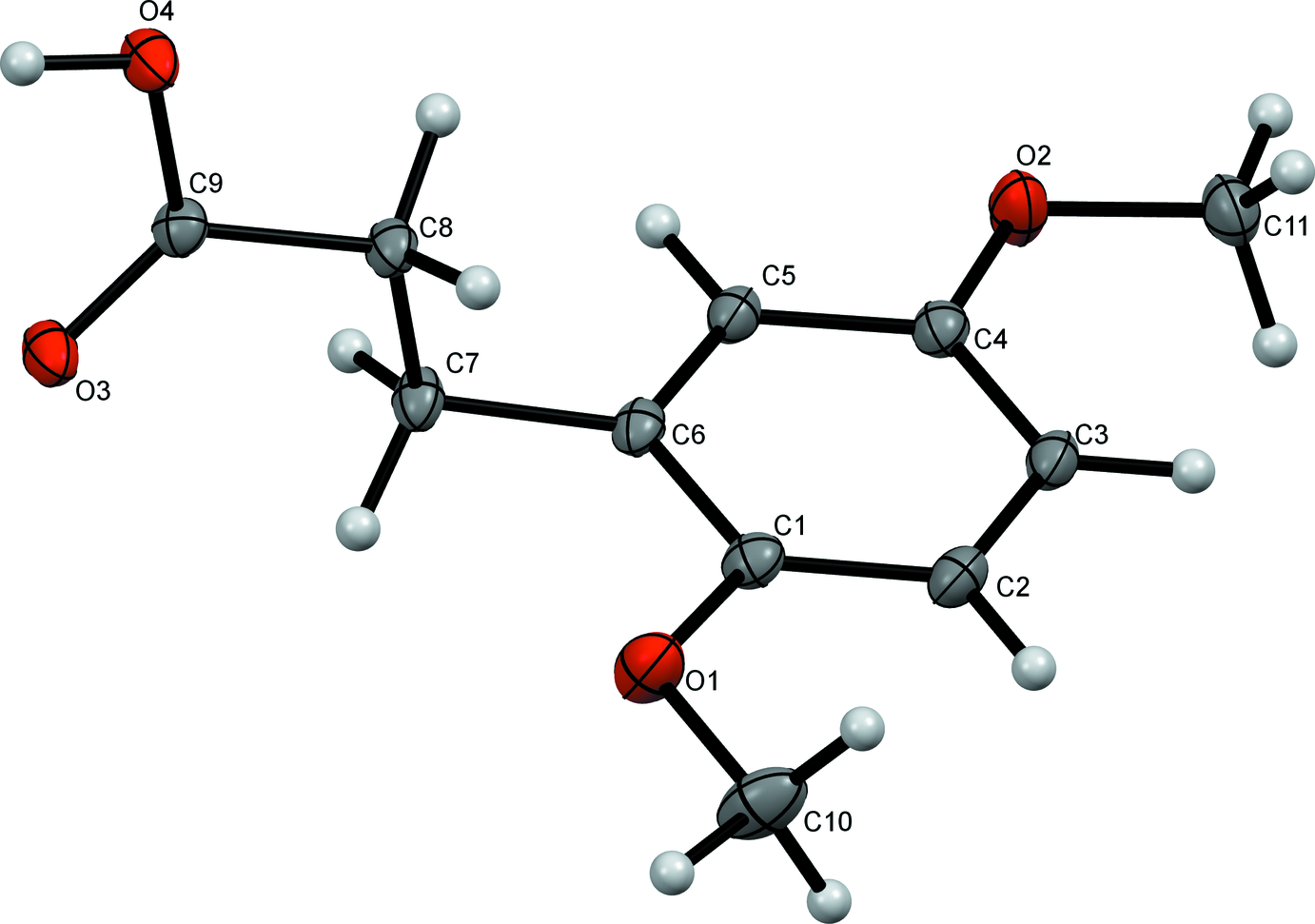

Supplement: Supplementary file 4 [file e-71-0o337-fig1.tif]
